# Zero-profile implant system versus novel plate systems after ACDF for comparison of sagittal balance parameters and clinical efficacy analysis

**DOI:** 10.1186/s13018-024-04857-y

**Published:** 2024-06-19

**Authors:** Yan Gong, Hang Zhuo, Zelin Zhou, Zhaojun Cheng, Yanchi Gan, Jiahui He, Zefeng Song, Hao Liu, Yu Liu, De Liang, Xiaobing Jiang, Hui Ren

**Affiliations:** 1 The First Affiliated Hospital of Guangzhou University of Chinese Medicine, Guangzhou, 510405 China; 2 The Affiliated TCM Hospital of Guangzhou Medical University, Guangzhou, 510120 China; 3 Department of Medicine, Dalian University of Technology, Dalian, 116081 China; 4 The Second Affiliated Hospital of Guangzhou Medical University, Guangzhou, 510260 China

**Keywords:** Sagittal balance parameters, ACDF, Zero-P, ZEVO, Skyline

## Abstract

**Background:**

The zero-profile implant system (Zero-P) and conventional plates have been widely used in anterior cervical discectomy and fusion (ACDF) to treat cervical spondylosis. The purpose of this study was to compare the effects of the application of Zero-P and new conventional plates (ZEVO, Skyline) in ACDF on the sagittal imaging parameters of cervical spondylosis patients and to analyze their clinical efficacy.

**Methods:**

We conducted a retrospective study on 119 cervical spondylosis patients from January 2018 to December 2021, comparing outcomes between those receiving the Zero-P device (n = 63) and those receiving a novel conventional plate (n = 56, including 46 ZEVO and 10 Skyline plates) through ACDF. Cervical sagittal alignment was assessed pre- and postoperatively via lateral radiographs. The Japanese Orthopedic Association (JOA), Neck Disability Index (NDI), and visual analog scale (VAS) scores were recorded at baseline, after surgery, and at the 2-year follow-up to evaluate patient recovery and intervention success.

**Results:**

There were significant differences in the postoperative C0-C2 Cobb angle and postoperative sagittal segmental angle (SSA) between patients in the novel conventional plate group and those in the Zero-P group (*P* < 0.05). Postoperatively, there were significant changes in the C2‒C7 Cobb angle, C0‒C2 Cobb angle, SSA, and average surgical disc height (ASDH) compared to the preoperative values in both patient groups (*P* < 0.05). Dysphagia in the immediate postoperative period was lower in the Zero-P group than in the new conventional plate group (0% in the Zero-P group, 7.14% in the novel conventional plate group, *P* = 0.046), and the symptoms disappeared within 2 years in both groups. There was no statistically significant difference between the two groups in terms of complications of adjacent spondylolisthesis (ASD) at 2 years postoperatively (3.17% in the Zero-P group, 8.93% in the novel conventional plate group; *P* = 0.252). According to the subgroup analysis, there were significant differences in the postoperative C2‒C7 Cobb angle, C0‒C2 Cobb angle, T1 slope, and ASDH between the ZEVO group and the Skyline group (*P* < 0.05). Compared with the preoperative scores, the JOA, NDI, and VAS scores of all groups significantly improved at the 2-year follow-up (*P* < 0.01). According to the subgroup analysis, the immediate postoperative NDI and VAS scores of the ZEVO group were significantly better than those of the Skyline group (*P* < 0.05).

**Conclusion:**

In ACDF, both novel conventional plates and Zero-P can improve sagittal parameters and related scale scores. Compared to the Zero-P plate, the novel conventional plate has a greater advantage in correcting the curvature of the surgical segment, but the Zero-P plate is less likely to produce postoperative dysphagia.

## Introduction

Cervical spondylosis, which is predominantly rooted in the degeneration of cervical intervertebral discs, emerges from the compounded degeneration of these discs and secondary facet joint degeneration. This progression leads to the compression of critical structures such as dural sacs, the spinal cord, nerves, and blood vessels, culminating in significant limitations in cervical and shoulder mobility, as well as pain and numbness, thus profoundly impacting patient quality of life [1]. The shifting dynamics of contemporary lifestyles and occupational demands have precipitated a consistent rise in the incidence of cervical spondylosis, underscored by an alarming trend toward younger affected populations. This trend not only poses substantial socioeconomic challenges but also severely compromises individuals’ quality of life [2, 3]. As evidenced by previous investigations, an overwhelming majority (80–90%) of individuals demonstrate signs of disc degeneration by the age of 50, as detected via cervical spine MRI [4, 5]. Traditional conservative treatments for common manifestations such as nerve root and spinal cord cervical spondylosis yield suboptimal outcomes, thereby rendering surgical intervention a more effective remedy [6].

Anterior cervical surgeries, including anterior cervical discectomy and fusion (ACDF), anterior cervical corpectomy and fusion, and artificial disc replacement, have been instrumental in achieving substantial spinal cord decompression and favorable postoperative clinical outcomes [7–9]. Since its initial description in the 1950s, ACDF has evolved into a conventional treatment modality for short-segment cervical spondylosis, efficaciously alleviating spinal cord compression and reinstating the anatomical curvature of the cervical spine, thereby enhancing patient quality of life [10, 11]. Despite the diversity of internal fixation devices available, ranging from Zero-Profile systems to various conventional plate systems, ongoing research debates their comparative clinical efficacy. Furthermore, postoperative sagittal balance of the cervical spine is posited to be significantly correlated with clinical outcomes [12]. The Zero-Profile system is limited by its ease of operation, reduced operation time, minimal exposure, and diminished risk of damage to vital tissues such as the esophagus and trachea [13]. However, concerns have been raised regarding its limited fixation strength and challenges in maintaining the physiological anterior convexity of the cervical vertebrae [14]. In contrast, conventional plates have been shown to significantly enhance interbody fusion rates and stability, mitigate the risk of implant displacement or fusion subsidence, and more effectively preserve the sagittal balance of the reconstructed cervical spine. This study introduces ZEVO and Skyline as innovative plate options, diverging from the Zero-Profile system [15]. The Skyline Anterior Cervical Plate System provides versatile endosseous implants and instruments that provide a good surgical field of view, accommodating different anatomical features of the patient and optimizing the design of the titanium plate, which has been prebent anteriorly to reduce the need for further bending of the plate. The unique large implant window allows for better visualization of the vertebral body, endplates, and implant position. The versatility of the ZEVO anterior cervical system allows the surgeon to insert screws into the vertebral body with a greater angle of deflection and adjust the guides to fit the desired angle within the system's range. The aim of this research was to retrospectively assess the enhancement of sagittal balance and long-term clinical efficacy following ACDF by employing various novel plates versus the Zero-P system in cervical spondylosis patients. Through comparative and subgroup analyses of these novel plate systems, this study aimed to determine the optimal anterior surgical approach for patients with cervical spondylosis, aiming to achieve maximal improvements in cervical sagittal balance and clinical outcomes.

## Materials and methods

### Participants

A retrospective analysis of 119 patients with cervical spondylosis who underwent ACDF between January 2018 and December 2021 was performed, including 63 patients treated with the Zero-P® (Johnson and DePuy Synthes, Shanghai, China) zero-profile cervical anterior cervical interbody fusion fixation system and 56 patients treated with anterior fixation systems using conventional plates. The new conventional plates used were ZEVO® (Medtronic, Shanghai, China) for 46 patients and Skyline® (Johnson & DePuy Synthes, Shanghai, China) for 10 patients. The inclusion criteria for patients were as follows: ① had a clear diagnosis of cervical spondylosis in ≤ 3 segments; ② had ineffective long-term medication, physical therapy or other treatments that seriously affected daily life; and ③ had all ACDFs, including the Zero-P, Skyline and ZEVO anterior cervical internal fixation systems. The exclusion criteria for patients were as follows: ① congenital deformity of the cervical spine; ② trauma or injury to the neck; ③ > 3 ossified segments of the posterior longitudinal ligament; ④ long-term oral aspirin, clopidogrel or other medications; ⑤ serious underlying disease (e.g., cardio-cerebral or pulmonary disease), inability to tolerate the surgery; and ⑥ history of previous cervical spine surgery. The study protocol was approved by the Clinical Trial Review Committee of the First Affiliated Hospital of Guangzhou University of Chinese Medicine (NO. K [2020] 104), and written informed consent was signed by all participants before the study. Patients in this study consented to the publication of their images.

### Surgical procedure: standard approach used in the Smith–Robinson technique

**Fig. 1:**
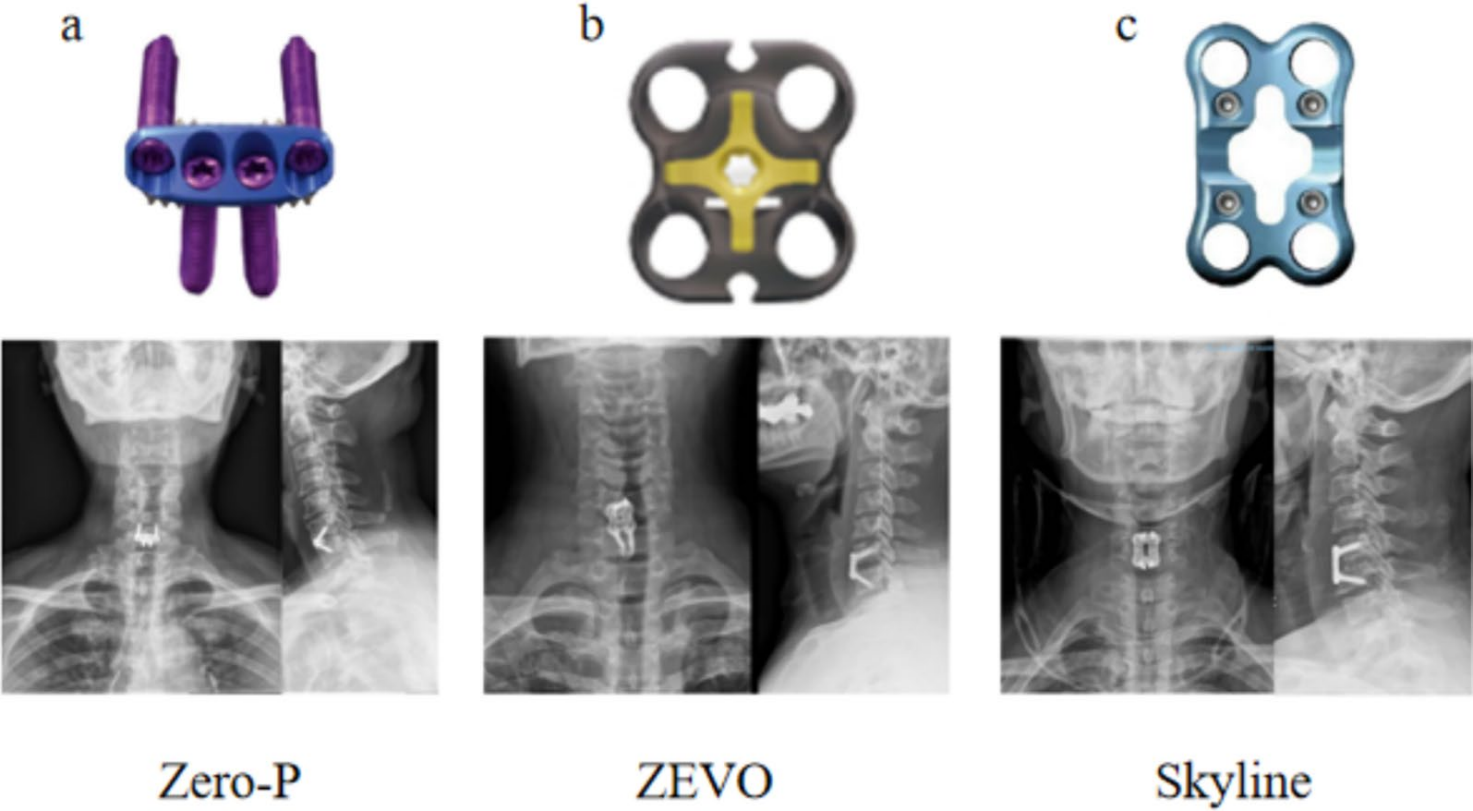
Schematic of the Zero-P® (**a**), ZEVO® (**b**), and Skyline® (**c**) implants and related postoperative X-ray images

**Fig. 2:**
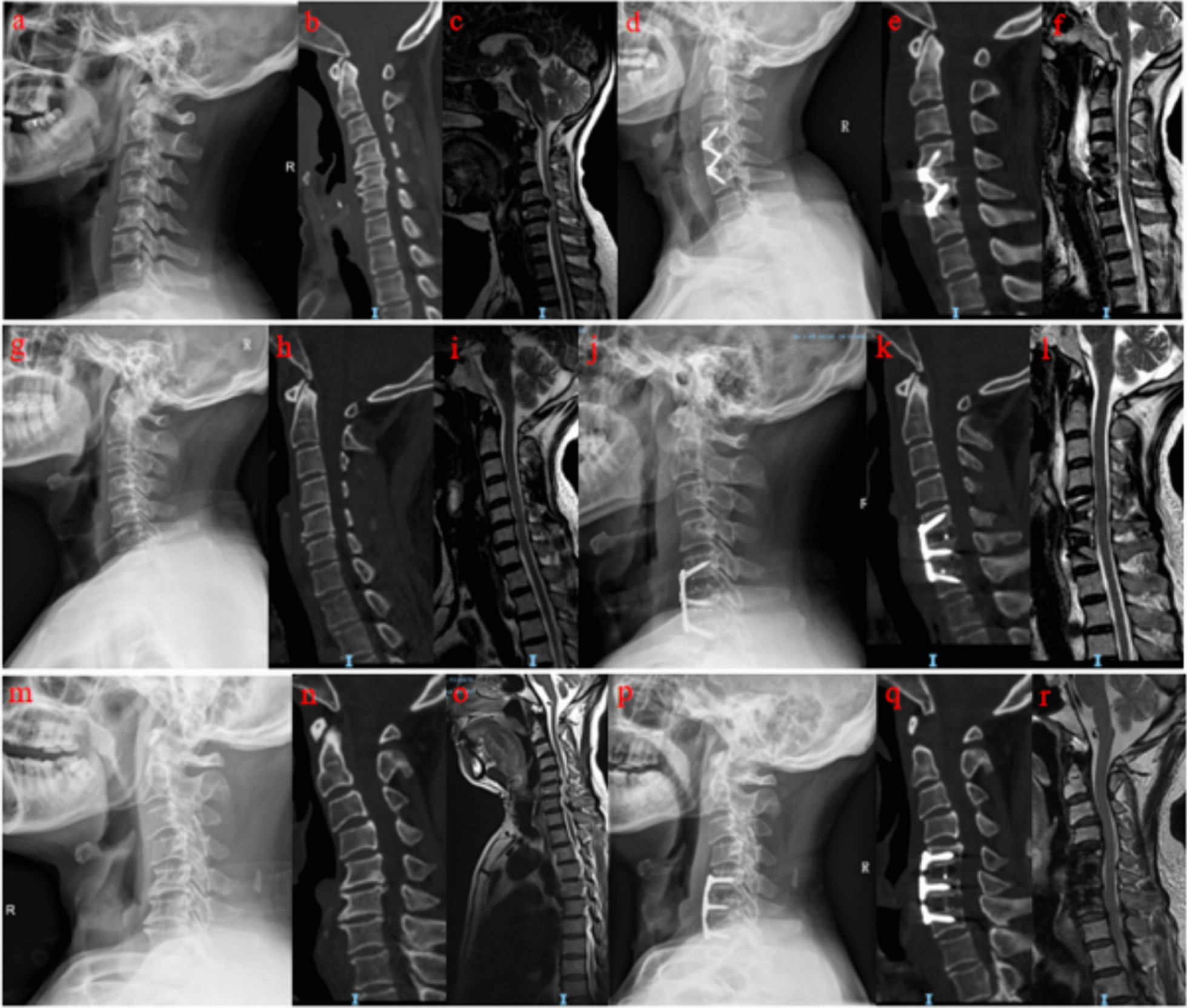
**a**–**f**: Pre- and postoperative radiographs of a 58-year-old woman following C4–C6 ACDF with the Zero-P system showing significant spinal improvement and decompression. **g**–**l** Post-C5-7 ACDF with the ZEVO System. Imaging of a 51-year-old man revealed resolved herniation and the absence of significant stenosis postsurgery. **m**–**r**: The efficacy of the Skyline System was demonstrated in a 71-year-old man, with pre- and postoperative images showing corrected alignment and decompression at C5–C7

The procedures were performed by the same attending surgeon and his team, and the patients were instructed to push the tracheal maneuver to the right side preoperatively to prevent excessive intraoperative tracheal pulling or discomfort caused by prolonged surgical time. Under general anesthesia, the patient was placed in the supine position, and after anesthesia, a transverse incision was made on the right side of the anterior cervical region after incising the cervical latissimus dorsi muscle and then between the carotid artery sheath and the visceral sheath. X-ray fluoroscopy was used to determine the accuracy of the surgical segments, and the spacer screw and spacer were implanted in the target vertebral body. The posterior longitudinal ligament of the target vertebral space was excised, all prolapsed disc tissue into the spinal canal was removed, and dural pulsation was observed after adequate decompression. The size of the fusion device was confirmed by filling the intervertebral space with a trial mold of the fusion device, and the fusion device was driven into the intervertebral space after the autogenous bone was placed into the fusion device and the plate was fixed with screws. Schematic diagrams of the different endoprosthetic fixation systems of the plate are shown in Fig. 1. Following the placement of the plate and screws, the C-arm X-ray machine was utilized once more for fluoroscopy to verify the correct positioning of the screws. Subsequently, the surgical area was thoroughly rinsed, and meticulous hemostasis was achieved using an electrocautery knife. The drains were then strategically placed to facilitate fluid removal. The surgical site was meticulously sutured, sterilized, and subsequently dressed with sterile gauze to maintain asepsis. Figure 2 presents the imaging data from three patients, each of whom underwent the application of distinct internal fixation systems.

### Radiographic analyses and data collection

**Fig. 3:**
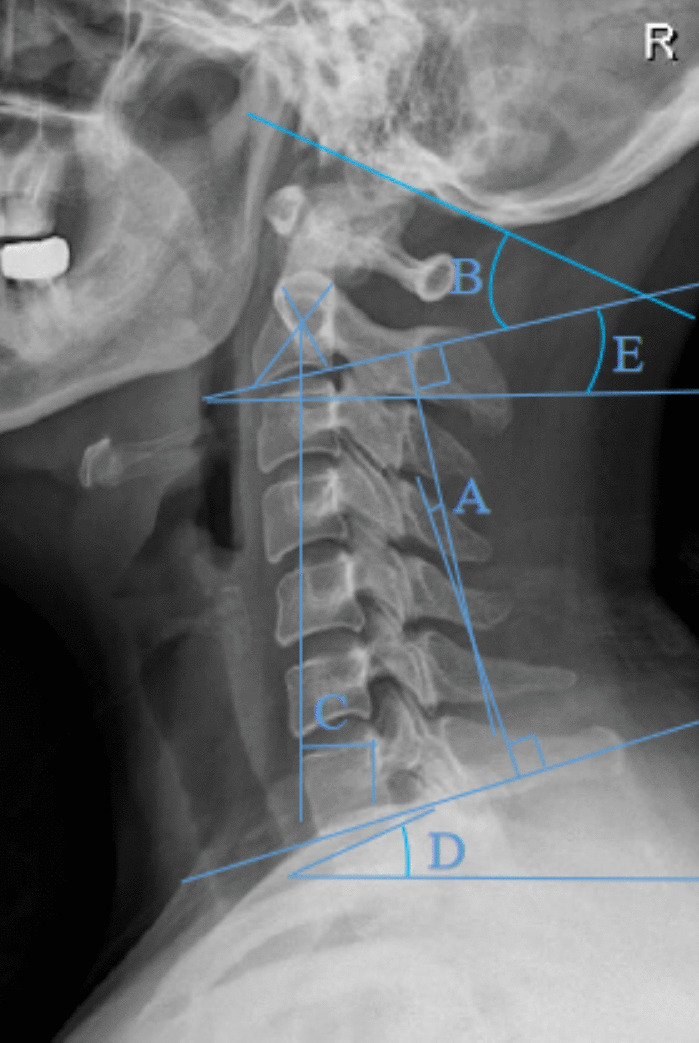
Radiographic measurements. **A** C2‒C7 Cobb angle. **B** C0‒C2 Cobb angle. **C** C2–C7 Sagittal Vertical Axis (SVA). **D** T1 slope. **E** C2 slope

The data collected included patient age, sex, surgery duration, intraoperative blood loss, and length of hospital stay for both groups. Preoperative and immediate postoperative lateral cervical spine radiographs, including flexion–extension images, were obtained to measure the parameters of cervical sagittal alignment. The C2‒C7 Cobb angle was defined as the angle between the vertical lines parallel to the inferior endplates of the C2 and C7 vertebrae. The C0‒C2 Cobb angle was measured as the angle between McRae's line and the inferior endplate of C2. The C2–C7 sagittal vertical axis (SVA) was determined by the distance from the plumb line extending from the center of C2 to the posterior-superior angle of C7 [16]. The T1 slope was defined as the angle between a horizontal line and the upper endplate of T1 [17]. The C2 slope is between the horizontal line and a straight line parallel to the lower end plate of C2 [18], and the specific measurements of the above metrics are shown in Fig. 3. The segmental alignment angle was the angle between the upper endplate of the upper vertebral body and the lower endplate of the lower vertebral body of the operated segment (SSA), the intervertebral height of the operated segment was the average of the anterior, middle, and posterior heights of the intervertebral space (ASDH), and we also collected the mobility of the upper and lower vertebral bodies of the operated segments in cervical power radiographs (upper and lower-SSA-ROM). Neurological function was assessed using the Japanese Orthopedic Association (JOA) [19] score for cervical spine conditions, while the severity of axial symptoms was quantified via the Neck Disability Index (NDI, 0 = no disability, 50 = total disability) [20]. Postoperative pain levels were evaluated using a visual analog scale (VAS) score [21]. JOA, NDI, and VAS scores were collected preoperatively, immediately postsurgery, and at the final follow-up, with a senior physician reviewing the outcomes. The surgical duration and amount of intraoperative blood loss were analyzed retrospectively. Diagnostic criteria for dysphagia after anterior cervical spine surgery [22]: the presence of the following symptoms of dysphagia for at least 3 weeks after surgery: ① swallowing dysfunction at the time of eating (dysphagia in swallowing dry, liquid, or bulky food, weakness, choking, etc.); ② discomfort at the time of swallowing (choking on a foreign body, burning sensation, etc.). Subsidence of the interbody fusion is diagnosed when a decrease in disc height of more than 3 mm in the operated segment compared to the immediate postoperative radiograph is observed at follow-up [23]. The imaging diagnosis of adjacent segment degeneration (ASD) is determined by cervical spine radiographs as follows [24]: (1) newly formed or enlarged anterior osteophytes; (2) increased intervertebral space narrowing; and (3) calcification of the anterior longitudinal ligament.

### Statistical analysis

We analyzed the data using SPSS version 21.0. Continuous variables are shown as the mean ± standard deviation. Qualitative information was analyzed using the chi-square test. Paired t tests were used to evaluate significant differences between preoperative and postoperative parameters. Statistical comparisons between the cervical sagittal parameters and various types of scores between the Zero-P and conventional plate groups (and between subgroups) were performed using the independent samples *t* test. *P* values less than 0.05 were considered to indicate statistical significance.

## Results

### Patient demographics

A total of 119 patients with ACDF were ultimately included: 63 patients in the Zero-P group, with a mean age of 51.57 ± 1.29 years, and 46 patients in the ZEVO group and 10 patients in the Skyline plate group, with a mean age of 55.91 ± 1.68 years. There were no significant differences between the two groups of the Zero-P group and the novel plate group in terms of sex, operative duration, intraoperative bleeding, range of motion (ROM), mobility of the upper segment of the operated segment (Upper-SSA-ROM), or mobility of the lower segment of the operated segment (Lower-SSA-ROM) (*P* > 0.05) (Table 1).

**Table 1:** Demographic and clinical data of the different groups

Parameters	Zero-P, n = 63	Plate (Skyline and ZEVO), n = 56	*P*
Age	51.57 ± 1.29	55.91 ± 1.68	0.040
Sex (male/female)	25/38	21/35	0.956
Operation time (min)	35.24 ± 34.87	34.46 ± 24.49	0.890
Blood loss (mL)	127.22 ± 35.72	127.20 ± 31.26	0.997
ROM (°)	32.72 ± 18.34	32.23 ± 14.49	0.869
Upper-SSA-ROM (°)	8.68 ± 4.28	8.71 ± 6.20	0.979
Lower-SSA-ROM (°)	9.50 ± 5.53	9.29 ± 4.43	0.820

### Radiographic results of the Zero-P group and novel conventional plate (Skyline and ZEVO) group

Preoperatively, except for the T1 slope (*P* < 0.01), there was no statistically significant difference in any of the other preoperative baseline indices between the patients in the Zero-P group and those in the novel plate group (*P* > 0.05). There was a significant difference between the C0-C2 cobb and SSA of the two groups of patients in the postoperative period (*P* < 0.05), and the novel plate was able to better restore the cervical curvature of the operated segments relative to the Zero-P group. Compared with those in the preoperative period, the C2–C7 Cobb angle, C0–C2 Cobb angle, SSA, and ASDH were significantly different between the two groups after surgery (*P* < 0.01). The T1 slope of the Zero-P group was significantly greater in the immediate postoperative period than in the preoperative period (*P* < 0.01), and the C2 slope was lower in the immediate postoperative period than in the preoperative period (*P* < 0.01) (Table 2). To compare the parameters between Zero-P and the plate more effectively, we also divided the patients into single-segment ACDF and multisegment ACDF groups for comparison (2 ≤ egments ≤ 3), and the results showed that there was no statistically significant difference between the two groups for any of the parameters in the patients who underwent single-segment and multisegment ACDF after the operation (Tables 3 and 4).

**Table 2:** Comparison of the sagittal parameters between the Zero-P group and the plate (Skyline & ZEVO) group

Parameters	Zero-P, n = 63	Plate (Skyline and ZEVO), n = 56	*P*
C2–C7 Cobb (°)			
Pre-operation	8.36 ± 6.27	8.08 ± 9.02	0.848
Post-operation	12.80 ± 8.15**	14.20 ± 9.92**	0.400
C0–C2 Cobb (°)			
Pre-operation	31.08 ± 6.96	29.02 ± 9.89	0.189
Post-operation	21.97 ± 5.77**	25.35 ± 10.28**	0.032
SVA (mm)			
Pre-operation	20.57 ± 11.51	20.50 ± 11.98	0.970
Post-operation	19.61 ± 9.56	21.10 ± 11.81	0.451
T1 slope (°)			
Pre-operation	19.54 ± 6.92	23.53 ± 8.42	0.005
Post-operation	25.40 ± 7.32**	25.13 ± 8.47	0.853
C2 slope (°)			
Pre-operation	12.14 ± 7.46	12.54 ± 6.48	0.757
Post-operation	9.62 ± 6.34**	11.26 ± 5.66	0.140
SSA (°)			
Pre-operation	5.45 ± 3.61	6.32 ± 6.12	0.356
Post-operation	7.29 + 4.67**	9.84 ± 6.53**	0.016
ASDH (mm)			
Pre-operation	5.84 ± 1.06	5.86 ± 1.25	0.924
Post-operation	9.28 ± 1.05**	8.84 ± 1.42**	0.058

SVA, Sagittal vertical axis; SSA, sagittal segmental angle; ASDH, average surgical disc height

**Compared with the preoperative values of the same group, the values were significantly different (*P* < 0.01)

*Compared with the preoperative value of the same group, significantly different, *P* < 0.05

**Table 3:** Single-segment ACDF: Comparison of the sagittal parameters between the Zero-P group and the plate (Skyline & ZEVO) group

Parameters	Zero-P, n = 37	Plate (Skyline and ZEVO), n = 12	*P*
C2–C7 Cobb (°)			
Pre-operation	9.37 ± 5.95	6.55 ± 7.43	0.186
Post-operation	12.66 ± 7.30*	13.62 ± 10.24**	0.772
C0–C2 Cobb (°)			
Pre-operation	30.80 ± 7.05	29.94 ± 10.09	0.742
Post-operation	22.10 ± 5.58**	26.02 ± 8.87	0.076
SVA (mm)			
Pre-operation	22.75 ± 10.13	22.58 ± 16.64	0.966
Post-operation	20.22 ± 9.78	23.60 ± 14.30	0.361
T1 slope (°)			
Pre-operation	19.96 ± 6.88	21.92 ± 8.88	0.428
Post-operation	23.97 ± 7.48**	24.38 ± 10.02	0.881
C2 slope (°)			
Pre-operation	12.69 ± 7.90	13.01 ± 6.76	0.900
Post-operation	10.24 ± 5.76*	12.22 ± 4.34	0.281
SSA (°)			
Pre-operation	5.22 ± 3.32	5.93 ± 5.52	0.594
Post-operation	5.67 + 3.32	6.10 ± 5.75	0.749
ASDH (mm)			
Pre-operation	5.88 ± 1.22	5.61 ± 1.29	0.508
Post-operation	9.38 ± 1.09**	8.92 ± 1.47**	0.246

SVA, Sagittal vertical axis; SSA, sagittal segmental angle; ASDH, average surgical disc height

**Compared with the preoperative values of the same group, the values were significantly different (*P* < 0.01)

*Compared with the preoperative value of the same group, significantly different, *P* < 0.05

**Table 4:** Multi-segment ACDF (2 ≤ egments ≤ 3): Comparison of the sagittal parameters between the Zero-P group and the Plates (Skyline and ZEVO) group

Parameters	Zero-P, n = 26	Plate (Skyline and ZEVO), n = 44	P
C2–C7 Cobb (°)			
Pre-operation	6.93 ± 6.56	8.50 ± 9.43	0.457
Post-operation	13.01 ± 9.37**	14.36 ± 9.95**	0.576
C0–C2 Cobb (°)			
Pre-operation	31.47 ± 6.95	28.77 ± 9.94	0.227
Post-operation	21.78 ± 6.12**	25.16 ± 10.71*	0.145
SVA (mm)			
Pre-operation	17.48 ± 10.46	19.93 ± 10.54	0.350
Post-operation	18.70 ± 9.34	20.42 ± 11.12	0.517
T1 slope (°)			
Pre-operation	18.94 ± 7.06	23.97 ± 8.35	0.012
Post-operation	27.44 ± 6.70**	25.34 ± 8.12	0.269
C2 slope (°)			
Pre-operation	11.34 ± 6.84	12.41 ± 6.47	0.519
Post-operation	8.74 ± 7.09	11.00 ± 5.99	0.159
SSA (°)			
Pre-operation	6.05 ± 4.39	6.42 ± 6.33	0.792
Post-operation	9.81 + 5.37*	10.98 ± 6.44**	0.451
ASDH (mm)			
Pre-operation	5.77 ± 0.81	5.93 ± 1.24	0.575
Post-operation	9.14 ± 1.00**	8.82 ± 1.43**	0.311

SVA, Sagittal vertical axis; SSA, sagittal segmental angle; ASDH, average surgical disc height

**Compared with the preoperative values of the same group, the values were significantly different (*P* < 0.01)

*Compared with the preoperative value of the same group, significantly different, *P* < 0.05

### Radiographic results of the Skyline and ZEVO models

Except for C0–C2 cobb (*P* < 0.01), there was no statistically significant difference in any of the other preoperative baseline indices between the two groups of patients in the Skyline group and the ZEVO group (*P* > 0.05). There were significant differences in postoperative C0–C2 cobb, postoperative Tl slope and ASDH between the two subgroups (*P* < 0.05). Postoperative C2‒C7 cobb, postoperative SSA and postoperative ASDH were significantly greater in the ZEVO group than in the preoperative period (*P* < 0.01), while postoperative C0‒C2 cobb was significantly greater in the Skyline group than in the preoperative period (*P* < 0.05). (Table 5).

**Table 5:** Subgroup comparison of the sagittal parameters of the plate group (Skyline versus ZEVO)

Parameters	Skyline, n = 10	ZEVO, n = 46	*P*
C2–C7 Cobb (°)			
Pre-operation	5.15 ± 13.44	8.72 ± 7.80	0.259
Post-operation	10.98 ± 11.81	14.90 + 9.47**	0.260
C0–C2 Cobb (°)			
Pre-operation	39.52 ± 8.63	26.74 ± 8.65	0.000
Post-operation	31.91 ± 9.26*	23.92 ± 10.01	0.025
SVA (mm)			
Pre-operation	16.46 ± 8.36	21.37 ± 12.53	0.243
Post-operation	17.81 ± 5.26	21.81 ± 12.72	0.119
Tl slope (°)			
Pre-operation	19.63 ± 7.65	24.38 ± 8.42	0.107
Post-operation	20.36 ± 8.56	26.17 ± 8.18	0.048
C2 slope (°)			
Pre-operation	13.64 ± 7.14	12.29 ± 6.39	0.555
Post-operation	11.37 ± 4.49	11.24 ± 5.93	0.948
SSA (°)			
Pre-operation	7.28 ± 5.65	6.11 ± 6.26	0.588
Post-operation	10.74 ± 5.60	9.65 ± 6.76**	0.636
ASDH (mm)			
Pre-operation	6.30 ± 1.18	5.76 ± 1.25	0.220
Post-operation	9.70 ± 1.79	8.65 + 1.28**	0.033

CSVA, Cervical sagittal vertical axis; SSA, sagittal segmental angle; ASDH, average surgical disc height

**Compared with the preoperative values of the same group, the values were significantly different (*P* < 0.01)

*Compared with the preoperative value of the same group, significantly different, *P* < 0.05

### Comparison of functional outcomes between the Zero-P and plate groups

**Fig. 4:**
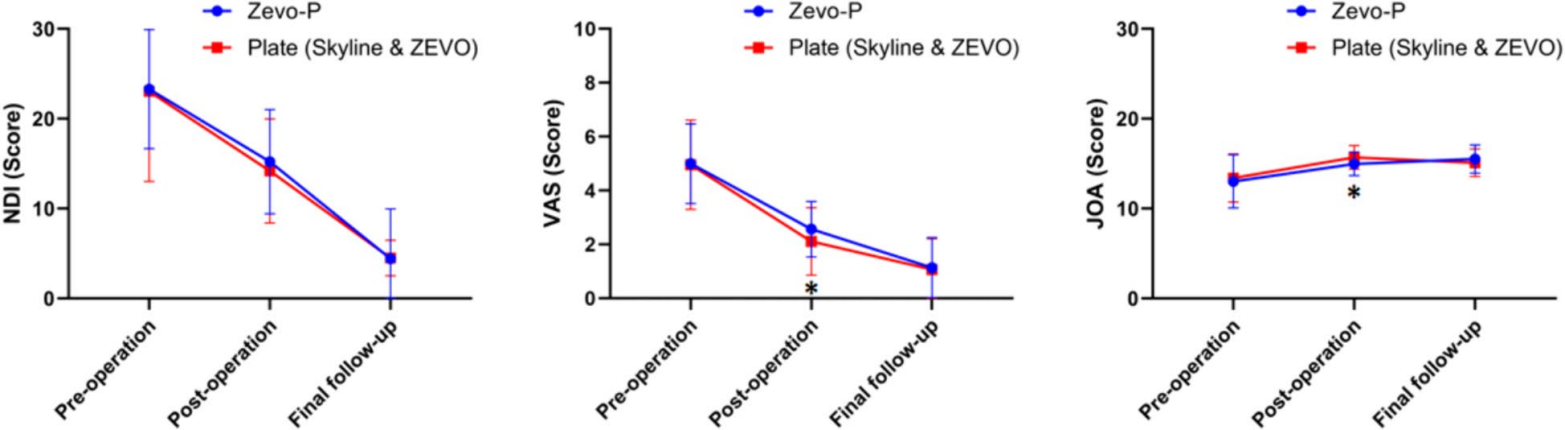
Comparisons of the NDI, VAS score and JOA score between the Zero-P group and the plate (Skyline & ZEVO) group at different time points. **Significantly different, *P* < 0.01. *Significantly different, *P* < 0.05

Observational analysis of the Zero-P group and the novel plate group revealed that the two groups of patients had significantly different immediate postoperative VAS and JOA scores (*P* < 0.05). However, there was no statistically significant difference in the NDI, VAS, or JOA score between the two groups at the final follow-up (*P* > 0.05), indicating that the two internal fixation materials were comparable for patient recovery at the final follow-up. When comparing the NDI and VAS scores at different time intervals, there was a statistically significant difference between the same groups in the immediate postoperative period and at the final follow-up visit compared with the preoperative period (*P* < 0.01). The JOA scores in the Zero-P group were significantly greater than both the preoperative and postoperative scores at the final follow-up (*P* < 0.01), whereas the JOA scores in the novel conventional plate group at the final follow-up, although greater than the preoperative scores (*P* < 0.01), decreased compared with the postoperative scores (*P* < 0.01) (Fig. 4). In patients who underwent single-segment ACDF, there was no significant difference in the NDI, VAS, or JOA score between the two groups in the immediate postoperative period or at the final follow-up (*P* > 0.05). Among patients who underwent multisegmental ACDF, there was a significant difference in the VAS and JOA scores between the two groups of patients in the Zero-P group and the new conventional plate group in the immediate postoperative period (*P* < 0.05). (Tables 6 and 7).

**Table 6:** Single-Segment ACDF: Comparisons of the NDI, VAS score and JOA score between the Zero-P group and the Plates (Skyline & ZEVO) group at different time points

Score	Zero-P, n = 37	Plate (Skyline & ZEVO), n = 12	*P*
JOA (score)			
Pre-operation	12.92 ± 3.03	14.42 ± 1.51	0.108
Post-operation	15.05 ± 1.37	15.50 ± 1.09	0.311
Final follow-up	15.35 ± 1.60**	15.33 ± 1.61	0.973
NDI (score)			
Pre-operation	23.24 ± 7.46	24.42 ± 9.82	0.664
Post-operation	14.97 ± 5.75	13.58 ± 2.28	0.421
Final follow-up	4.43 ± 2.70**	4.42 ± 1.44**	0.985
VAS (score)			
Pre-operation	4.78 ± 1.72	5.08 ± 1.56	0.595
Post-operation	2.46 ± 0.93	2.17 ± 0.84	0.337
Final follow-up	1.30 ± 1.18**	1.00 ± 1.21**	0.453

**Comparison of preoperative values with those of the same group at the final follow-up; significantly different, *P* < 0.01

*Comparison of preoperative values with those of the same group at the final follow-up; significantly different, *P* < 0.05

**Table 7:** Multisegment ACDF (2 ≤ egments ≤ 3): Comparisons of the NDI, VAS score and JOA score between the Zero-P group and the Plates (Skyline and ZEVO) group at different time points

Score	Zero-P, n = 26	Plate (Skyline and ZEVO), n = 44	*P*
JOA (score)			
Pre-operation	13.15 ± 2.92	13.11 ± 2.86	0.955
Post-operation	14.85 ± 1.22	15.75 ± 1.38	0.008
Final follow-up	15.73 ± 1.54**	15.02 ± 1.53**	0.066
NDI (score)			
Pre-operation	23.35 ± 5.34	22.66 ± 10.16	0.751
Post-operation	15.54 ± 5.98	14.34 ± 6.43	0.443
Final follow-up	4.35 ± 2.40**	4.50 ± 2.12**	0.781
VAS (score)			
Pre-operation	5.31 ± 1.01	4.93 ± 1.70	0.310
Post-operation	2.73 ± 1.15	2.09 ± 1.34	0.047
Final follow-up	0.92 ± 0.98**	1.09 ± 1.14**	0.532

**Comparison of preoperative values with those of the same group at the final follow-up; significantly different, *P* < 0.01

*Comparison of preoperative values with those of the same group at the final follow-up; significantly different, *P* < 0.05

### Subgroup comparison of functional outcomes between the Skyline group and the ZEVO group

**Fig. 5:**
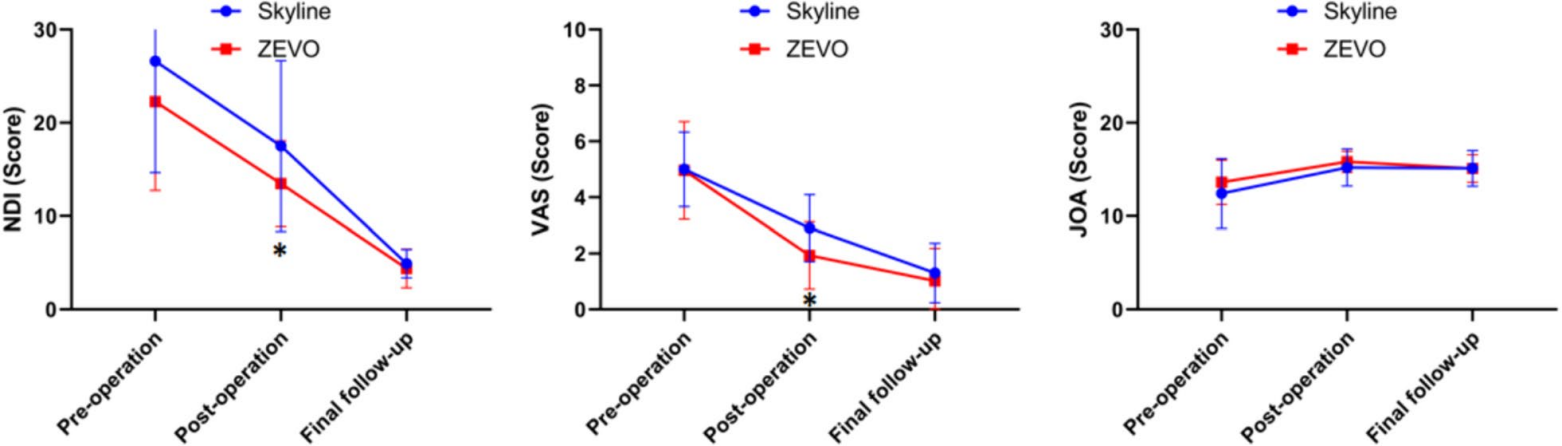
Subgroup comparisons of the NDI, VAS score and JOA score between the Skyline group and ZEVO group at different time points. ** Significantly different, *P* < 0.01. * Significantly different, *P* < 0.05

In the observational analysis of the Skyline group and the ZEVO group, there was a statistically significant difference in the NDI and VAS scores between the two groups in the immediate postoperative period (*P* < 0.05). There was no statistically significant difference between the two groups in the immediate postoperative JOA scores and the NDI, VAS, and JOA scores at the final follow-up (*P* > 0.05). There was a statistically significant difference in the NDI, VAS, and JOA scores between the Skyline group and the ZEVO group at different time intervals and between the same groups in the immediate postoperative period and at the final follow-up compared with the preoperative period (*P* < 0.01) (Fig. 5).

### Complications

Four patients in the plate group (7.14%, 4/56) and 0 patients in the Zero-P group developed dysphagia postoperatively, and there was a statistically significant difference between the two groups (χ^2^ = 4.657, *P* = 0.046). Their severity was mild, and none of the patients had dysphagia at the final follow-up. Two patients (3.17%, 2/63) in the Zero-P group developed ASD in the adjacent vertebrae, whereas five patients (8.93%, 5/56) in the plate group developed ASD, and there was no statistically significant difference between the two groups (χ^2^ = 1.773, *P* = 0.252). The other patients recovered well after surgery without further symptoms. No cases of implant failure, fusion subsidence, plate or screw loosening, or dislocation were observed in any patient.

## Discussion

In recent years, with the increase in the use of mobile devices and electronic products, cervical spondylosis due to prolonged neck flexion has led to changes in anatomical curvature, cervical spine load increases and accelerated cervical spine degeneration, and the incidence of cervical spondylosis has also increased [25–27]. It has been demonstrated that patients' cervical spinal space and disc herniation can improve with the restoration of the cervical lordosis angle [26], and when the cervical curvature straightens and, in more severe cases, posterior bowing occurs, there is an imbalance in the cervical sagittal parameters, allowing for narrowing of the intervertebral space, disc herniation and compression of the spinal cord.

The sagittal balance of the cervical spine is receiving increasing attention from spine surgeons [28]. The human body's daily life depends on the normal curvature of the cervical spine, and when its curvature changes, the biomechanical equilibrium is disrupted, which in turn accelerates the onset and development of cervical spine degeneration and causes cervical spondylosis [29]. There is a vicious cycle between cervical sagittal imbalance and cervical spine degeneration. In addition, there is a physiological anterior convex angle in the cervical spine, which is the structure with the largest range of motion of the spinal column, and when the anterior convexity disappears and even the cervical spine recoils due to poor posture or excessive strain, the center of the head moves forward, the arm of force in the cervical spine activities relative to the center of rotation of the cervical increases, and the muscle ligament of the posterior cervical area as well as facet joints compensate for doing the work, which in turn causes intervertebral joints to hyperostosis and a series of degenerations to occur [30]. Cervical instability due to cervical degenerative disease can also lead to cervical sagittal imbalance, causing changes in cervical spine biomechanics, which in turn leads to neurological damage and loss of physiologic curvature [31]. ACDF has been proven to be a classic procedure for the treatment of short-segment, nerve-root and spinal cord cervical spondylosis. The procedure is safe, has fewer complications, is less traumatic, involves fewer steps, and is a very standardized process that is easy for spine surgeons to learn and master [32]. By removing compressed dural sac tissues such as intervertebral discs and hyperostosis in the target responsible segment, the procedure can fully decompress the spinal cord and nerve roots, and by combining fusion implantation with traditional plate screw internal fixation and the Zero-P anterior internal fixation system, the impaired nerve function and cervical anatomical curvature can be restored, and intervertebral fusion can be used to restore the height of the intervertebral space. There is growing evidence that cervical sagittal parameters play a key role in determining the appropriate surgical access for patients undergoing cervical spine surgery [33]. There have also been finite element studies demonstrating the effect of ACDF on the physical dynamics of sagittal kyphosis and cervical fusion in adjacent segments [34]. The anterior cervical internal fixation system used in ACDF can effectively restore the local sagittal sequence of the cervical spine as well as the intervertebral height and has definite clinical efficacy [35].

Surgical treatment of cervical spondylosis should not only focus on adequate decompression of the spinal nerves of the diseased segments but also emphasize reconstruction of the local sagittal alignment [36], and the cervical lordosis curvature is usually taken as an important reference for assessing the equilibrium status of the cervical sagittal alignment. The results of this study showed (Tables 2 and 3) that there were statistically significant differences in the C2–C7 Cobb angle, C0–C2 Cobb angle, SSA, and ASDH between the Zero-P group and the novel plate group of internal fixation systems compared with the preoperative period, with the C2–C7 Cobb angle, as the main part of the cervical anatomical curvature, showing greater improvement in cervical curvature in the immediate postoperative period, and the ASDH increased, which was in line with the results of previous studies [26]. Although there are more studies on the C0–C2 Cobb angle and atlantoaxial subluxation, there are fewer reports on the effect of this angle on the preoperative and postoperative period of ACDF because the alignment of the upper and lower cervical vertebrae and the sagittal balance of the cervical spine need to be viewed as a whole [37, 38], and we included this metric to explore whether it would have an effect on the sagittal balance of the cervical spine of patients who underwent ACDF. Mutual adaptation and compensation between the C0–C2 Cobb angle and the C2–C7 Cobb angle in the maintenance of cervical posture and cervical movement have been found [39], and a negative correlation between the two changes has been demonstrated in healthy populations [40]. Matsunaga et al. concluded that for patients who undergo occipitocervical fusion surgery, the C0–C2 Cobb angle needs to be controlled to 0°–30° to minimize the long-term postoperative effects on the middle and lower cervical spine [41]. This suggests that too large a C0–C2 Cobb angle has some effect on the anatomical curvature of the cervical spine, and we found that the patients included in this study all had large C0–C2 Cobb angles, and all of them significantly improved after surgery. In the Zero-P group versus the novel plate group, the postoperative C0–C2 Cobb angle was reduced and significantly improved compared with that in the preoperative period according to both the same-group comparison and the two-group comparison. Similarly, Dohzono et al. reported increased motion of the atlanto-occipital joints after laminoplasty [42], and Xiao et al. reported that a tendency toward compensatory changes and degeneration of the occipito-atlanto-axial vertebral complex may lead to poorer clinical outcomes, suggesting that compensatory changes in upper cervical curvature can be used to evaluate ACDF complications [37]. In the present study, for the upper cervical angle, all changes in the C0–C2 Cobb angle were reduced from the preoperative period, which may reduce postoperative complications. Subgroup analysis of conventional plates in this study was performed because we considered that the structure of different types of plates may have an effect on the sagittal balance of the cervical spine. There was already a significant preoperative difference in the C0–C2 Cobb angle in the subgroups, with the Skyline group showing greater improvement in the C0–C2 Cobb angle and improvements in the C2–C7 Cobb angle, the SSA, and the ASDH being more pronounced in the ZEVO group; however, the probable reason for this is the smaller number of samples in the Skyline group that we included. Anderst and Phillips suggested that maintaining the cervical lordosis angle and cervical mobility have different meanings and that there is an interaction between them, with compensatory changes occurring [37, 39]. In a prospective study of single-segment ACDF, increased curvature of postoperative surgical segments was found to improve postoperative pain and disability-related scores [39]. In this study, both the Zero-P group and the novel plate group showed significant improvement in postoperative SSA compared with the preoperative period, and the novel conventional plate group showed better improvement than did the Zero-P group. Among the subgroups, the ZEVO group showed better improvement than did the Skyline group.

Both the novel plate group and the Zero-P group showed a significant increase in the mean intervertebral space height after surgery compared with the preoperative period, indicating that both surgical procedures significantly increased the intervertebral height. Moreover, the mean postoperative interbody height in the Skyline plate group was significantly greater than that in the ZEVO plate group according to the subgroup analysis. This may be related to the unique large-implant window design of the Skyline plate, which allows for more adequate intervertebral implantation to achieve the greatest possible intervertebral height while facilitating visualization of the vertebral body, endplates, and implant position. Postoperative disc height is negatively correlated with neck pain, with smaller disc heights associated with increased neck pain [43]. However, we should not aim for an excessive increase in the intervertebral space, as it has been reported that excessive distraction of the intervertebral disc space in a cadaveric model leads to an increase in contact pressure between the graft and the cervical endplate [44]. The study confirmed that changes in intervertebral space height could be considered a predictor of postoperative dysphagia after single-segment ACDF, and when the change in intervertebral space height was ≥ 3 mm, the odds of postoperative dysphagia were significantly greater [45].

Although the clinical results of the anterior cervical plate screw internal fixation system have been affirmed, some common clinical complications have been gradually emphasized, such as postoperative screw loosening or extraction, fusion sinking, and screw and plate fracture, which can lead to life-threatening failure of internal fixation [46]. Different screw placement angles have different effects on the stress transfer effect, which determines the future stability of the internal fixation system and has clinical significance for long-term internal fixation failure and adjacent segment degeneration [47]. Clinical improvements in screw placement techniques can reduce the incidence of internal fixation failure of anterior cervical plate screws. MA et al. conducted an experimental study and determined that the optimal direction of screw placement is when the screw is placed into the vertebral body, when the upper screw is slightly inclined upward, when the lower screw is slightly inclined downward, and when the placed screw exhibits parallelogram mechanics, which significantly reduces the bending and folding shear force of the screw and improves its stability [48]. By biomechanical analysis of a single screw, SEEBECK et al. reported that screwing the screw at an angled angle increased the pullout strength of the screw [49]. In this study, they all used cross placement of screws at an angle to the endplate, which we found to improve the stability of internal fixation. We found through follow-up that there were no complications, such as screw loosening or fusion sinking, in this study.

Zhang et al. demonstrated that the use of both Zero-P and conventional plates in single-segment ACDF surgery was a safer and more effective strategy and that there was no statistically significant difference between the two groups of patients in terms of JOA, VAS, or NDI scores at the final postoperative follow-up [50]. A comparative study on the use of zero-p versus conventional plates in single-segment ACDF surgery revealed that the zero-p group had a shorter operative time, less intraoperative blood loss, a shorter follow-up JOA score, and a lower incidence of dysphagia and postoperative ASD [51]. The results of this study also confirmed that the Zero-P group was able to reduce the incidence of postoperative dysphagia. There was no statistically significant difference in the improvement rates of the JOA score, VAS score, or NDI between the Zero-P group and the novel conventional plate group in this study, indicating that the two surgical modalities had comparable efficacy in terms of patients' neurologic recovery, pain symptoms, or axial symptoms. This study confirmed that the Zevo plate significantly improved patients' immediate postoperative VAS and NDI scores compared to the Skyline plate group, but there was also no statistically significant difference between the two subgroups at the 2-year postoperative follow-up time point, suggesting that the two plates are comparable in terms of improving patients' postoperative outcomes in the long term.

This study has several limitations: (1) only patients with cervical spondylosis of ≤ 3 segments were included in this study, and only patients who underwent anterior surgery who underwent ACDF were included; (2) the different internal fixation systems still need to be further analyzed by finite element analysis or macroscopic specimen testing to determine the mechanisms involved in restoring the overall sagittal alignment of the cervical spine. (3) The number of patients included in the subgroups was low, and further expansion of the number of patients included is needed.

## Conclusion

In conclusion, ACDF employing both the Zero-P and novel plate systems facilitated improvements in cervical sagittal parameters and associated patient-reported outcomes. Notably, the use of conventional plates led to a more pronounced enhancement in the curvature of the surgical segment, which contributed to a comparatively better maintenance of cervical sagittal balance, but the Zero-P was less likely to produce postoperative dysphagia. Among the novel plates, ZEVO demonstrated superior immediate postoperative clinical outcomes compared to those of Skyline plates, although both were equally effective in enhancing long-term patient outcomes following surgery. However, this study is not without limitations, including its single-center nature, retrospective design, constrained dataset, and brief follow-up duration. To comprehensively evaluate and contrast postoperative sagittal balance modifications across both groups and subgroups, a multicenter study with an extended follow-up period is warranted.

## Data Availability

The data used to support the fndings of this study are included within the article.

## Abbreviations

Zero-P: Zero-profile implant system
ACDF: Anterior cervical discectomy and fusion
JOA: Japanese Orthopedic Association
NDI: Neck Disability Index
VAS: Visual Analogue Scale
SSA: Sagittal segmental angle
ASDH: Average surgical disc height
SVA: Sagittal vertical axis
